# Are EMS bypass policies effective implementation strategies for intravenous alteplase for stroke?

**DOI:** 10.1186/s43058-020-00041-5

**Published:** 2020-06-05

**Authors:** Alex H. S. Harris, Nicolas B. Barreto, Amber W. Trickey, Sylvia Bereknyei, Tong Meng, Todd H. Wagner, Prasanthi Govindarajan

**Affiliations:** 1 Department of Surgery, Stanford – Surgery Policy Improvement Research & Education Center (S-SPIRE), Stanford, CA 94305 USA; 2 Veterans Affairs Health Services Research and Development Center for Innovation to Implementation, Palo Alto Veterans Affairs Health Care System, Menlo Park, CA 94025 USA; 3 Department of Emergency Medicine, Stanford University, Stanford, CA USA

**Keywords:** Policy evaluation, Stroke treatment, CFIR, Mixed-methods

## Abstract

**Background:**

Stroke is a leading cause of disability and the fifth leading cause of death in the USA. Intravenous alteplase is a highly effective clot-dissolving stroke treatment that must be given in a hospital setting within a time-sensitive window. To increase the use of intravenous alteplase in stroke patients, many US counties enacted policies mandating emergency medical service (EMS) paramedics to bypass local emergency departments and instead directly transport patients to specially equipped stroke centers. The objective of this mixed-methods study is to evaluate the effectiveness of policy enactment as an implementation strategy, how differences in policy structures and processes impact effectiveness, and to explore how the county, hospital, and policy factors explain variation in implementation and clinical outcomes. This paper provides a detailed description of an Agency for Healthcare Quality and Research (AHRQ)-funded protocol, including the use of the Consolidated Framework for Implementation Research (CFIR) in the qualitative design.

**Methods/design:**

We will construct the largest-ever national stroke database of Medicare enrollees (~ 1.5 million stroke patients) representing 896 policy counties paired with 1792 non-policy counties, then integrate patient-, hospital-, county-, and state-level covariates from eight different data sources. We will use a difference-in-differences analysis to estimate the overall effect of the policy enactment on intravenous alteplase use (implementation outcome) as well as key patient outcomes. We will also quantitatively examine if variation in the context (urban/rural status) and variation in policy features affect outcomes. Finally, a CFIR-informed multiple case study design will be used to interview informants in 72 stakeholders in 24 counties to identify and validate factors that enable policy effects.

**Discussion:**

Policies can be potent implementation strategies. However, the effects of EMS bypass policies to increase intravenous alteplase use have not been rigorously evaluated. By learning how context and policy structures impact alteplase implementation, as well as the barriers and facilitators experienced by stakeholders responsible for policy enactment, the results of this study will inform decisions regarding if and how EMS bypass policies should spread to non-policy counties, and if indicated, creation of a “best practices” toolkit.

Contributions to the literature

- Without knowing the overall effectiveness of EMS bypass policies to increase use of intravenous alteplase, or the context and policy features that impact effectiveness, it is unknown if or how these policies should be spread to new counties.
- Policies are rarely conceptualized as implementation strategies and evaluated within an implementation science-informed mixed-methods research design.
- This AHRQ-funded protocol highlights an innovative use of a CFIR-informed, multiple case study qualitative design, along with triangulation from diverse data sources. Other researchers may find this example useful in designing rigorous mixed-methods policy evaluations.

## Background

This protocol paper provides a detailed description of an Agency for Healthcare Quality and Research (AHRQ)-funded protocol to evaluate if widely enacted county-level EMS bypass policies are effective implementation strategies to increase the reach of intravenous alteplase, a clot-dissolving medication for stroke patients. The protocol also provides a detailed description of how an implementation framework and methods can be used to inform the design, refinement, and evaluation of policy interventions as implementation strategies.

Among the 500,000 adults who suffer a stroke every year, 15% die within 30 days and 30% are still not functionally independent 90 days later [1]. Intravenous alteplase, a highly effective drug that dissolves clots to treat stroke, must be given in a hospital setting within a time-sensitive window [2–4]. This drug has been shown to lower mortality and improve both short- and long-term functional independence [2, 5–7]. However, lack of specialized stroke teams, rapid imaging, and care delays limits the number of patients who receive intravenous alteplase. To overcome these challenges, the “stroke center” designation was created to identify facilities equipped to provide acute stroke treatment including the timely administration of intravenous alteplase [8]. Early results from a national sample showed that intravenous alteplase use was almost two times higher at these stroke centers compared to non-stroke centers [9–11]. The higher intravenous alteplase use at stroke centers led to a primary stroke center certification program by The Joint Commission in 2004, and consequently, by 2011, more than 800 stroke centers had been established in the USA [12, 13].

However, not all stroke patients are transported by emergency medical service (EMS) to stroke centers. To extend the observed benefits of stroke centers to all stroke patients, state legislators, public health departments, and national organizations recommended building stroke systems of care in which EMS paramedics would bypass local emergency departments and directly transport patients to stroke centers (EMS bypass policy) [14]. The concept of EMS bypassing some emergency departments to deliver patients to facilities with specialized capacity was also supported by better patient outcomes observed in the cardiac and trauma systems of care. Following a few early adopters of stroke bypass policies, a majority of US counties enacted new bypass policies between 2007and 2011 [15–17]. However, the evaluation of the policies’ effects in the USA has been limited to single urban counties; has not controlled for secular trends or patient-, hospital-, county-, and state-level covariates; and has not assessed key stroke patient outcomes such as 30-day mortality and short- and long-term functional independence at 90 days and 1 year later [18–20]. Thus, it is unknown how effective bypass policies are for increasing alteplase use, or how context and policy structure impact alteplase implementation. Furthermore, the barriers and facilitators experienced by stakeholders responsible for policy enactment have not been examined to guide counties presently considering bypass policies. Therefore, overall goal of this study is to inform decisions regarding if and how bypass policies should spread to non-policy counties, and to develop best practice toolkits for future implementers.

## Research design and methods

### Overview

**Fig. 1:**
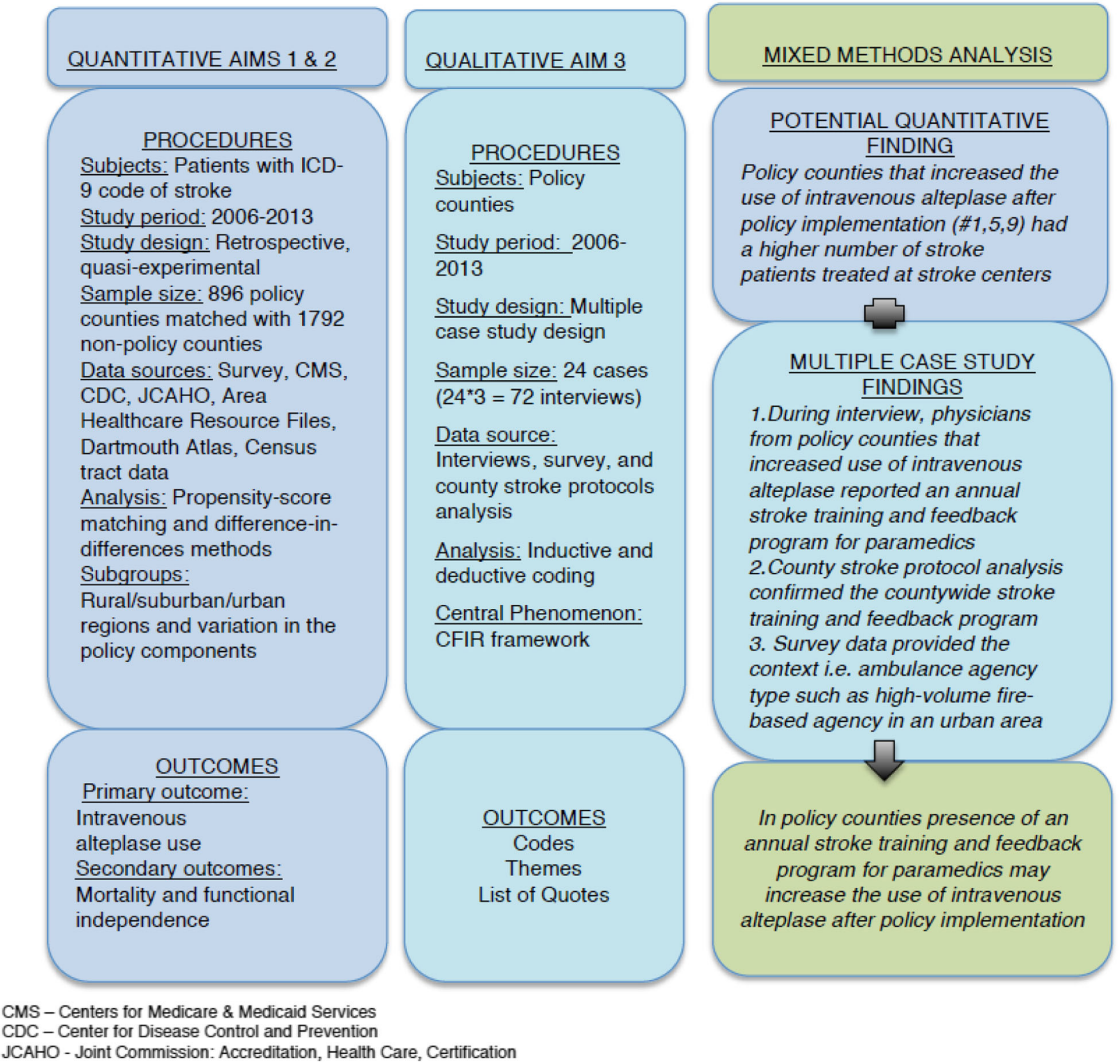
Explanatory sequential mixed-methods design with quantitative, qualitative, and mixed-methods analysis

This explanatory sequential mixed-methods proposal has three aims. In aim 1, we will test whether policy counties had higher intravenous alteplase use (implementation outcome) and better clinical stroke outcomes (lower 30-day mortality, and greater short- and long-term functional independence) than non-policy counties. In aim 2, we will test if urban counties had higher use of intravenous alteplase and better stroke patient outcomes than non-urban counties and if features of policy structure or implementation impacted outcomes. In aim 3, we will qualitatively explore the county- and hospital-level factors that enabled higher intravenous alteplase use and better stroke patient outcomes. Figure 1 depicts the overall structure of the project proposal.

### Theoretical models

The Andersen Model for Health Services Utilization [21] guides our quantitative evaluation of policy effects on intravenous alteplase use and resultant patient outcomes, which include 30-day mortality and 90-day and 1-year functional independence. We will control for multilevel covariates, e.g., policy variation at the state, ambulance use by stroke patients and urban/rural status at the county, stroke center or teaching status of the hospital, and age, sex, and race/ethnicity of patients. In aim 3, we will use Consolidated Framework for Implementation Research (CFIR) to guide interviews of stroke and EMS physician leaders and the paramedics. CFIR is a taxonomy of constructs thought to be related to variation in implementation success and downstream outcomes [22]. We will examine 13 CFIR constructs (described below) that we hypothesize to be relevant to policy effectiveness and intravenous alteplase implementation.

### Aims 1 and 2

Aim 1 tests if the county-level bypass policy led to higher intravenous alteplase use (implementation outcome), lower 30-day mortality, and greater 90-day and 1-year functional independence (clinical outcomes). Aim 2 tests whether policy effects varied by characteristics of the county.

#### Sample and analytic dataset

For aim 1, we will construct a database of Medicare enrollees with a stroke diagnosis during 2006–2013, using several different data sources (Table 1). In the building phase, we will add the policy and non-policy counties, enactment dates, and state-level policy types from emergency medical service state officials to the database. We will further enrich the database with hospital- and county-level covariates. Concurrently, we will prepare, validate, and distribute a survey to 896 counties with a bypass policy to collect data on ambulance, demographics, and policy components (see survey in Supplemental File A). Then, we will add the survey results to the database.

**Table 1:** Source and list of multilevel covariates to be used in aims 1 and 2 models

	CMS data	Stroke certification programs	American Hospital Association annual survey	Survey of 896 counties	Area healthcare resource files	US census data	Area deprivation index
Study outcomes							
Intravenous alteplase use	✓						
Mortality	✓						
Functional independence	✓						
Patient							
Age	✓						
Sex	✓						
Race/ethnicity	✓						
Comorbidities	✓						
Hospital							
Stroke center status		✓					
Teaching status			✓				
Hospital size	✓						
County							
Bypass components				✓			
Ambulance type				✓			
Ambulance use	✓						
Transport distance	✓						
Neurologist coverage					✓		
Urbanized areas	✓					✓	
Socio-economic status						✓	✓
State							
Policy type				✓			

#### Study setting and design

We will identify the month in which US counties adopted the policy between 2006 and 2013. If a state adopted a policy, then we will assign the implementation date to each county in the state. We will evaluate treatment during the baseline period represented by the calendar year prior to the policy implementation. Two post-policy periods will be analyzed, represented by the first and second calendar years following policy implementation. The statistical models will assess the pooled effect across multiple years of policy implementation. An indicator variable will represent the actual year of policy implementation to maintain accurate modeling of real-time effects. We will only include counties that implemented the policy during 2006–2013 since rapid expansion of the policy occurred during this period.

### Outcomes

Our primary implementation outcome is intravenous alteplase use among ischemic stroke patients abstracted from CMS data. Our secondary (clinical) outcomes are 30-day mortality and functional independence (short-term) at 90 days and (long-term) at 1 year. Intravenous alteplase treatment will be identified using documentation of ICD-9 procedure code 99.10—injection or infusion of thrombolytic agents—or the Current Procedure Terminology (CPT) codes for infusion of intravenous alteplase (37201) and infusion of the drug other than coronary (37202) and intravenous alteplase for stroke (37195). Presence of the ICD-9 procedure code or the CPT code has a sensitivity of 82% (95% CI 69–90%) and a specificity of 98% (95% CI 95–99%) as shown in recent studies [23, 24]. Our secondary outcomes of functional independence (time spent at home at 90 days and 1 year from first day of index stroke admission) have a strong correlation with the gold standard modified Rankin Scale [25]. The in-hospital mortality data will be combined with discharge to hospice variable to get the standard 30-day mortality data. These variables will be abstracted from CMS data.

#### Data analysis plan

We will employ a difference-in-differences analysis to evaluate the effect of stroke policy enactment on intravenous alteplase use, while accounting for the change in outcome over time in non-policy counties. The main implementation outcome will be intravenous alteplase use (yes/no). Mixed effects hierarchical logistic regression models with random intercepts for hospital, county, and state will be constructed to estimate the effect of stroke policy on intravenous alteplase treatment. Patient-level data (level 1) are naturally nested within hospitals (level 2), within counties (level 3), and within states (level 4).

### Model specification

(Aim 1) In the analytical phase, we will match the data from the non-policy counties with the data from policy counties (in the ratio of 2:1), using a propensity score method. The matching criteria include counties within the same geographic region and those with similar baseline intravenous alteplase use (parallel trends before policy implementation). In the final step, we will apply the difference-in-differences method to isolate the effect of the policies on outcomes using an interaction term for the policy by time period in mixed effects logistic regression models with random intercepts for clustering in hospitals, counties, and states.

We will extend aim 1 to consider possible variation across counties, especially the effects of urban/rural status or variation by policy components. Linking CMS data with the Census codes will identify the level of urbanization of the patient residence. Census block-level classification of urbanized areas is determined by the population density of interrelated geographic units. Urbanized areas and urban clusters consist of core Census block groups or blocks with population density of 1000 persons/sq. mile and surrounding Census blocks with 500 persons/sq. mile; urbanized areas have a population ≥ 50,000, urban clusters have a population of 2500–49,999, and rural areas make up the remaining non-urban blocks [26, 27]. For each county unit in the analysis, Census urbanization data will be aggregated to determine the percentage of the population living in each urbanization category. We will also examine whether the effect of the bypass policy is moderated by variation in policy components using the 3-way interaction terms as described above.

### Aim 3

To qualitatively explore the hospital and county factors that will explain the bypass policy’s effects, revealed in aims 1 and 2, on implementation and clinical outcomes.

#### Overview

In the context of our overall explanatory sequential mixed-methods design, we will identify the qualitative sample based on quantitative findings. The nested multiple case study sampling strategy be used to identify 24 policy counties (our “cases”) that will be systematically selected based on their intravenous alteplase treatment use (aims 1 and 2 results) and their urbanized area (urban), urban cluster (suburban), or rural status. Within each case, we will conduct three interviews of physicians and paramedics guided by the CFIR. The interview data will be combined with the aim 1 survey and document analysis (county stroke protocols) to triangulate data from multiple sources and identify themes that explain the policy’s effect on stroke treatment. This entire process of identifying themes and explanation-building using data from different counties will help to evaluate plausible explanation for the policy’s success as an implementation strategy. The qualitative results will be mixed with the quantitative findings using “matching” and presented as a joint display [28]. The output will also inform the development of a “best practices toolkit” for future policy enactment.

#### Multiple case study design [29]—sampling strategy and sample size

We will increase the county representation by using a purposive criterion sampling in which we will select “cases” by their intravenous alteplase use identified in by aims 1 and 2 results. This stratification will result in four groups: (1) high performers who remain high, (2) initially high performers that experience a drop in intravenous alteplase use, (3) low performers who remain low, and (4) low performers that increase intravenous alteplase use. We will determine the high performance as counties in the top 10th percentile for intravenous alteplase use and low performance as counties in the bottom 10th percentile for intravenous alteplase use. Within each of the four groups, we will sample two “cases” from each metropolitan area type (urbanized areas, urban clusters, and rural) resulting in a total of 24 counties (4 groups, 6 “cases”/group). Within each “case,” we will interview three providers (3 × 24 = 72 interviews of stroke physician leader, EMS physician leader, and a paramedic). Data from these interviews will be combined with aim 1 survey data and document analysis of the county’s stroke protocols to identify the factors that explain the policy effect.

#### Site informant selection and recruitment

Within each sampling group, we will create a list of “cases.” Starting at the top of each list, we will identify three key informants within each “case” focusing on county-wide “hot spots” such as paramedics who are responsible for triage decision to stroke centers and leadership who are responsible for the integration and communication between paramedics and the stroke centers. If the primary informants are unable to participate, we will recruit others from related services through key informant sampling [29]. They will be recruited through e-mail and then by phone. The initial e-mail will state the purpose of the study and highlight the “case” specific data pre-and post-policy. Participation will be voluntary, and participants will not be identifiable in the results. We anticipate that about three participants from each site over 24 sites will allow us to reach thematic saturation, the end point for qualitative data collection. However, this number is an estimate and the final number will be determined when thematic saturation occurs, as defined as a point in the study when new participants do not provide information that adds new codes or themes to the data. In the unlikely event that we are unable to recruit for the interviews, we will work our way down the ranked list until the sample size is reached or thematic saturation occurs.

#### Interview guide development

Table 2 presents the CFIR constructs that are applicable to this proposal and that will guide development of an open-ended, semi-structured interview guide. This semi-structured format allows for standardization across key questions and flexibility to clarify, probe, or expand on participants’ responses. We will perform cognitive testing of the interview questions and pilot-test with leaders from non-study counties. The interview will use CFIR constructs to probe into hospital factors such as presence of a medical director with stroke expertise and quality improvement process and provider factors such as staff education that are likely to explain the quantitative outcomes in aims 1 and 2 of the study [22]. CFIR constructs not central to this evaluation include trialability of the intervention (e.g., pilot-testing policy is not feasible at county-level) and implementation climate (e.g., limited recall of training programs in early implementation).

**Table 2:** CFIR constructs that will guide aim 3 interview questions

Hospital characteristics	
Organizational structure	Number of beds, stroke center, and academic status
Leadership engagement	Medical director with stroke knowledge/expertise
Communication network within an organization	Stroke teams, labs and diagnostic radiology 24/7, communication with the emergency department
Provider characteristics	
Knowledge	Attitudes towards the policy
Outer setting	
Networking with other organizations	Get with the guidelines membership, collaboration with the local ambulance agencies
Process	
Evaluation	Quality improvement activities

We will conduct and audio-record the interviews with participant’s consent, using the secure video-conferencing facility provided by the communication portals used by the Stanford Health Care.

#### Survey

We will use existing data from the aim 1 survey to provide ambulance demographic information such as type of ambulance agency, transport volume, and agency composition for the qualitative sample. This will help compare and contrast the cases’ ambulance resources and their influence on the policy’s effect. We will explore the responses to the open-ended questions on the reasons for the policy implementation and triangulate this with the interview findings and document analysis to strengthen the evidence.

#### Document analysis

In the qualitative sample, we will collect and review the county stroke protocols for paramedics during the study period. We will catalog policy components such as use of stroke scale and training, bypass time, and routing time, and will provide necessary context and elements to further characterize policy counties with high and low intravenous alteplase use. The document analysis findings will be triangulated for confirmatory/disconfirmatory evidence with the interview findings and survey data to identify best practices within and across counties and county groups.

#### Qualitative analysis

We will use a professional service to transcribe the interview content and will conduct the analysis facilitated by NVivo software (version 11). Each transcript will be reviewed for clarity and quality before being finalized for coding and analysis. Our qualitative expert will read, identify patterns in the transcripts, and develop a codebook. Initial codes will reflect our research questions and CFIR domains (deductive), and subsequent codes will encompass unexpected or new themes that emerge from the data (inductive). Each code will be assigned a detailed definition, and subject to both inclusion and exclusion criteria. To ensure inter-coder agreement, two investigators will double code the first two transcripts, assess areas of divergence, and finalize the codebook. The investigators will also perform inter-rater reliability test on the consistency of code application across the codebook, with a minimum expectation of pooled Cohen’s kappa 0.7. We will then concurrently conduct interviews and code with the final stable codebook so that subsequent interviews can elicit richer information on emergent themes.

#### Mixed-methods analysis

Using an explanatory sequential design, the qualitative data collection will be “matched” to complement the quantitative data collection. This readily facilitates analysis through a joint display as the quantitative results and the qualitative themes can be linked in a single table where the findings are juxtaposed together. Within NVivo, the unit of organization will be the individual county to match the quantitative data. As the data emerge, we will be able to compare the data quantitative across the analyst-defined subgroups such as rural, urbanized areas, and urban clusters. The construction of joint displays enhances both analysis and presentation of mixed-methods data. This integrates the qualitative and quantitative data in a mixed-methods analysis where the mixed-methods purpose will be draw “metainferences,” i.e., conclusions based on consideration of the qualitative and quantitative data together.

## Study status

Dataset construction and survey and interview protocol refinement and pilot testing are underway.

## Discussion

Without knowing the overall effectiveness of stroke bypass policies to increase use of intravenous alteplase, or the context and policy features that impact effective, it is unknown if or how these policies should be spread to new counties. The purpose of this AHQR-funded study is to answer these questions within an implementation science-informed mixed-methods research design. Beyond documenting the overall methods of this study, this protocol paper highlights an innovative use of a CFIR-informed, multiple case study qualitative design, along with triangulation from diverse data sources. We hope other researchers find this example useful in designing other rigorous mixed-methods policy evaluations.

Often policy evaluators and implementation scientists exist in different silos, each with their own terminologies and methods. For example, the difference-in-differences analytic framework proposed in this study is common in policy evaluation and health economics, but uncommon in implementation research. Conversely, CFIR is foundational in implementation science but rarely utilized by in policy evaluations. Policies are rarely conceptualized as implementation strategies for specific healthcare interventions and evaluated with an implementation science lens. Our multidisciplinary team of investigators with expertise in emergency medicine, statistics, health economics, qualitative and mixed-methods design, and implementation science attempted to create a study capable of answering the core questions and to inform consideration of future bypass policy enactments.

## Supplementary information

**Additional file 1:.** Supplemental File A

## Data Availability

Not applicable
