# Supplementary material for: Are EMS bypass policies effective implementation strategies for intravenous alteplase for stroke?
Source: Implement Sci Commun. 2020 Jun 5;1:50. doi: 10.1186/s43058-020-00041-5 (PMC7427915; doi:10.1186/s43058-020-00041-5)
Supplement: Supplementary file 1 — Additional file 1:. Supplemental File A [file 43058_2020_41_MOESM1_ESM.docx]

One-Tier Bypass Policy Evaluation (ACT & TREAT) – FINAL

**CONSENT**

Emergency Medical Service Leaders,

You are invited to participate in a research study examining county-level stroke bypass policies that direct EMTs (emergency medical technician; including basic, intermediate, and paramedic) to bypass local emergency departments and transport patients to thrombolysis-capable centers (referred to as one-tier regionalization policy). This study seeks to determine if one-tier regionalization policies have led to higher intravenous alteplase use, lower 30-day mortality, and greater short-and long-term functional independence. This study is funded by the Agency of Healthcare Research and Quality, 1R01HS026207-01A1.

As a part of this study, we are asking you to answer this survey to help us understand when, how, and why a stroke bypass policy was implemented in the county (or counties) you oversee. The survey also collects some descriptive information about your county. Please feel free to consult any colleagues or documents you need to complete this survey.

The expected benefits of this study include better estimates of the effects of one-tier regionalization policies and insight into the factors that affect effective policy implementation. You will receive no payment for your participation.

There are no anticipated risks associated with this study. Your participation will not be disclosed so there will be no adverse effects to your status or employment.

Your participation is voluntary, and you have the right to withdraw your consent or discontinue participation at any time without penalty. You also have the right to refuse to answer particular question(s). The results of this research study may be presented at scientific or professional meetings or published in scientific journals. Your individual privacy will be maintained in all published and written data resulting from the study. If you have any questions, concerns, or complaints about this research, its procedures, risks, or benefits, contact the Protocol Director, Prasanthi Govindarajan, at (650) 498-3700. If you have any technical or administrative questions, please contact Michael Vernon at (650) 721-2450.

If you are not satisfied with how this study is being conducted, or if you have any concerns, complaints, or general questions about the research or your rights as a participant, please contact the Stanford Institutional Review Board (IRB) to speak to someone independent of the research team at (650)-723-5244 or toll free at 1 (866) 680-2906. You can also write to the Stanford IRB, Stanford University, 3000 El Camino Real, Five Palo Alto Square, 4th Floor, Palo Alto, CA 94306.

You may print a copy of this page for your records.

By clicking on “I agree”, you agree to participate in the study and fully understand the content of this information sheet.

- I agree
- I disagree

**COVERAGE AREA**

**State**

“What state do you work in? (select from dropdown list)”

▼Alabama ... Wyoming

**County Count**

“In how many counties do you serve as Medical Director of Emergency Medical Services?”

▼1 ... 15

**Email**

“Please provide an email address where you can be reached:”

________________________________________________________________

**Phone**

“Please provide a phone number where you can be reached:”

________________________________________________________________

**COUNTY DETAILS**

“Please list the name(s) of county(ies) you oversee below, and whether they have implemented the one-tier regionalization policy.

We are only interested in collecting information from counties that have implemented a one-tier regionalization policy; i.e. EMTs *currently* bypass local emergency departments and transport patients to thrombolysis-capable centers. Therefore, if none of the counties in your coverage area has implemented (i.e. EMTs *do not* bypass local emergency departments), you will be directed to the end of the survey.”

|  | County Name | Policy implemented? | |
| --- | --- | --- | --- |
|  |  | Yes, Implemented | No, Not Implemented |
| County 1 |  |  |  |
| County 2 |  |  |  |
| County 3 |  |  |  |
| County 4 |  |  |  |
| County 5 |  |  |  |
| County 6 |  |  |  |
| County 7 |  |  |  |
| County 8 |  |  |  |
| County 9 |  |  |  |
| County 10 |  |  |  |
| County 11 |  |  |  |
| County 12 |  |  |  |
| County 13 |  |  |  |
| County 14 |  |  |  |
| County 15 |  |  |  |

**COUNTY DIFFERENCES**

The following question was only visible to participants who oversee multiple counties.

“Did the policies implemented in the counties you serve have different components (i.e. public campaigns, screening tools, etc.)?”

- Yes
- No **COUNTY POLICIES - SIMILAR**

The following question was only visible if participants indicated they oversee multiple counties, and if those counties initiated *similar* policies.

“You have indicated that, in the counties you serve, the policies implemented had similar components. 

When the policies started (EMTs started to bypass local emergency departments) which of these components were implemented? 

Please check all that apply:”

- Stroke public awareness campaigns
- Stroke screening tool used by EMTs
- Notification of incoming stroke patient
- "Last seen normal" time limits (bypass decision based on time since patient was last seen normal)
- "Maximum routing" time limits (bypass decision based on rerouting time)
- Designated stroke centers to administer intravenous alteplase for acute stroke
- Other (please specify): ________________________________________________

**COUNTY INVOLVEMENT**

Participants only answered about the counties that they listed above that *had a policy*. County names were pipped in from the above question.

“Of the county(ies) you serve that have completed one-tier regionalization policy implementation, were you directly involved in the implementation process?”

|  | Involvement | |
| --- | --- | --- |
|  | Yes | No |
| County name 1 |  |  |
| County name 2 |  |  |
| County name 3 |  |  |
| County name 4 |  |  |
| County name 5 |  |  |
| County name 6 |  |  |
| County name 7 |  |  |
| County name 8 |  |  |
| County name 9 |  |  |
| County name 10 |  |  |
| County name 11 |  |  |
| County name 12 |  |  |
| County name 13 |  |  |
| County name 14 |  |  |
| County name 15 |  |  |

**COUNT SPECIFIC QUESTIONS**

Below is the list of questions asked about each county that the participant indicates they oversee and *have implemented* a policy.

**Q#.1**

“Please answer the following questions about the one-tier regionalization policy implemented in *county name pipped in from above* County.

As a reminder for this survey, a "one-tier regionalization" policy is one that directs EMTs to bypass the local emergency departments and transport patients to thrombolysis-capable centers.”

**Q#.2**

“Was the one-tier regionalization policy mandated by the state?”

- Yes
- No

**Q#.3**

“Was the one-tier regionalization policy mandated by the county?”

- Yes
- No

**Q#.4**
“What date did the policy implementation process *start* (EMTs started to bypass local emergency departments) in *county name pipped in from above* County?

(If you don’t know the exact date, please answer as best you can)”

| Month | ▼ January (1) ... (12) |
| --- | --- |
| Day | ▼ (1) ... (31) |
| Year | ▼ (1900) ... (2049) |

**Q#.5**

Participants answer this question for each county *only if* they indicate that the counties they oversee have different policies.

“When the policy was *started* (EMTs started to bypass local emergency departments), which of these components were implemented in *county name pipped in from above* County? 

Please check all that apply:”

- Stroke public awareness campaigns
- Stroke screening tool used by EMTs
- Notification of incoming stroke patient
- "Last seen normal" time limits (bypass decision based on time since patient was last seen normal)
- "Maximum routing" time limits (bypass decision based on rerouting time)
- Designated stroke centers to administer intravenous alteplase for acute stroke
- Other (please specify): ________________________________________________

**Q#.6**

“Are you aware of any quality improvement processes built into the one-tier regionalization policy implemented in *county name pipped in from above* County (e.g. Audit and feedback loop, technical assistance, etc.)?”

- Yes
- No

**Q#.7**

“Please answer the following questions about how *county name pipped in from above* County implemented the one-tier regionalization policy.

Recall that "policy implementation" refers to when EMTs actively bypassing local emergency departments.”

**Q#.8**

“When the one-tier regionalization policy planning began, did *county name pipped in from above* County have a relationship with any of the following? 

Please check all that apply:”

- American Heart/Stroke Association (e.g. Get With The Guidelines Program)
- Paul Coverdell Registry
- State EMS Authority
- National EMS Organizations
- Peer EMS Agencies
- Other (please specify): ________________________________________________

**Q#.9**

“Please indicate which of the following agencies were involved in the one-tier regionalization planning process in *county name pipped in from above* County?

Please check all that apply:”

- EMS agency administration
- Hospital administration
- Other (please specify); ________________________________________________

**Q#.10**

“Were you aware of any of the following external (outside of the county) reasons to implement the policy in *county name pipped in from above* County? 

Please check all that you were aware of:”

- State recommendations, but not requirements
- Bench-marking by external agencies
- National stroke guidelines
- Monetary incentives
- Other (please specify):________________________________________________

**Q#.11**
“Please answer yes or no to the following questions.

Before EMTs started bypassing local emergency departments, during the planning process in *county name pipped in from above* County…”

|  | Yes | No |
| --- | --- | --- |
| There was an external agent or “champion” facilitating the implementation |  |  |
| Someone was formally appointed to lead the implementation |  |  |

**Q#.12**

“Were there adequate resources to implement the one-tier regionalization policy in *county name pipped in from above* County?”

- Yes
- No

**Q#.13**

“When a one-tier regionalization policy was implemented in *county name pipped in from above* County (EMTs began to bypass emergency departments), were any of the following informational resources available?

Please check all that apply:”

- Tool kits/resource binders
- Onsite consultants
- External consultants
- Online resources
- Other, please specify ________________________________________________

**Q#.14**

“Was there any resistance when planning the implementation of the one-tier regionalization policy in *county name pipped in from above* County?”

- Yes
- No

**Q#.15**

Participants only answer this question if they indicate there was resistance to the policy.

“You indicated there was some resistance to the one-tier regionalization policy in *county name pipped in from above* County. 

Was the resistance internal (within the county administration) or external (outside of the county administration)?”

- Internal
- External
- Both

**Q#.16**

“Please rate the extent to which you agree or disagree with the following statements. Recall that "implementation" refers to when EMTs started bypassing local emergency departments. 

When the one-tier regionalization policy was implemented in *county name pipped in from above* County...”

|  | Strongly agree | Somewhat agree | Neither agree nor disagree | Somewhat disagree | Strongly disagree |
| --- | --- | --- | --- | --- | --- |
| ...I thought there was enough evidence to implement the policy. |  |  |  |  |  |
| ...I thought the policy would improve thrombolysis treatment. |  |  |  |  |  |
| ...I thought the policy would improve patient functional independence. |  |  |  |  |  |
| ...I thought the policy would be a general improvement in stroke care. |  |  |  |  |  |
| ...I thought the practice used before the policy had similar outcomes. |  |  |  |  |  |

**Q#.17**

“The following questions are about *county name pipped in from above* County in general. 

Recall that "policy implementation" refers to when EMTs began to bypass local emergency departments.”

**Q#.18**

“What type of ambulance agency served *county name pipped in from above* County at the time the one-tier policy was implemented? 

Please check all that apply:”

- Fire-based agency
- Hospital-based agency
- Community based paid agency
- Community based volunteer agency
- Private/commercial agency
- Third service agency
- Critical care agency
- Air medical transport
- Other (please specify): ________________________________________________

**Q#.19**

“What was the composition of the ambulance agency(ies) in *county name pipped in from above* County at the time of policy implementation?”

Please check all that apply:”

- EMT Basic Technicians
- EMT Intermediate Technicians
- EMT Paramedics
- Critical care nurses
- Physicians

**Q#.20**

“Was there a county EMS medical director in *county name pipped in from above* County at the time of policy implementation?”

- Yes
- No
- Don't know

**Q#.21**

“Which stroke center criteria are used by ${state/ChoiceGroup/SelectedAnswers}and/or *county name pipped in from above* County?

Please check all that apply:”

- External certifying body
- State criteria
- County criteria

**Q#.22**

“Since the one-tier policy began, have any changes or additions to the policy been made in *county name pipped in from above* County?”

- Yes
- No

**Q#.23**

Participants only answer the following question if they indicate that changes have been made to the policy.

“You indicated that additions to the one-tier policy were made after it began in *county name pipped in from above* County. 

What additions were they? Please check all that apply.”

- Stroke public awareness campaigns
- Stroke screening tool used by EMTs
- Notification of incoming stroke patient
- "Last seen normal" time limits (bypass decision based on time since patient was last seen normal)
- "Maximum routing" time limits (bypass decision based on additional time to stroke center)
- Designated stroke centers to administer intravenous alteplase for acute stroke
- Other (please specify): ________________________________________________

**Q#.24**

“Are you willing to provide a copy of the one-tier policy that was implemented in *county name pipped in from above* County? If so, please enter the best contact email below:”

_____________________________________________

**SUBJECTIVE QUESTIONS**

Participants only answer the following questions after completing all the county level questions about counties that have the policy.

“The following questions are about your experience as a leader of the implementation of a one-tier policy.

Please rate the extent to which you agree or disagree with the following statements:”

|  | Strongly agree | Somewhat agree | Neither agree nor disagree | Somewhat disagree | Strongly disagree |
| --- | --- | --- | --- | --- | --- |
| I learned a lot from the policy implementation process. |  |  |  |  |  |
| I was in charge of the policy implementation. |  |  |  |  |  |
| I was enthusiastic about the policy implementation. |  |  |  |  |  |
| I was committed to the policy implementation. |  |  |  |  |  |
| The policy implementation process was complex. |  |  |  |  |  |
| Implementing the policy was a team effort. |  |  |  |  |  |

**EARLY END OF SURVEY QUESTION**

Participants only answer this question if they *do not* *oversee* any counties that have a one-tier regionalization policy.

“You have indicated that the county(ies) you serve have not implemented a one-tier regionalization policy, so you are not eligible to complete this survey. 


Before exiting the survey, do you have any additional comments/thoughts on one-tier regionalization policies? If so, please enter below:”

________________________________________________________________

________________________________________________________________

________________________________________________________________

________________________________________________________________

________________________________________________________________
